# Prognostic significance of pyroptosis-associated molecules in endometrial cancer: a comprehensive immunohistochemical analysis

**DOI:** 10.3389/fonc.2024.1359881

**Published:** 2024-03-18

**Authors:** Seong-Chan Ha, Yeon Soo Park, Jisup Kim

**Affiliations:** ^1^ Gachon University College of Medicine, Incheon, Republic of Korea; ^2^ Department of Pathology, Gil Medical Center, Gachon University College of Medicine, Incheon, Republic of Korea

**Keywords:** pyroptosis, endometrial cancer, NLRP3, cleaved caspase-1 p20, cleaved gasdermin D, CHMP4B, recurrence-free survival, overall survival

## Abstract

**Introduction:**

Endometrial cancer, the most prevalent malignancy of the female genital tract, has a concerningly poor prognosis when diagnosed in advanced stages, with limited targeted therapy options available for advanced or recurrent cases. Pyroptosis, a type of nonapoptotic cell death mediated by caspase-1, has shown potential antitumor effects in various tumors. NLRP3, a cytosolic sensor, initiates the canonical pyroptotic pathway, leading to caspase-1 activation, subsequent gasdermin D cleavage, and plasma membrane pore formation. The ESCRT-III machinery, particularly CHMP4B, acts as a key inhibitor of pyroptosis by repairing gasdermin D-induced membrane damage. The current study aimed to evaluate the clinicopathologic relevance of key pyroptosis-associated molecules in endometrial cancer.

**Methods:**

Immunohistochemistry was used to assess the expression of four pyroptosis-associated molecules (NLRP3, cleaved caspase-1 p20, cleaved gasdermin D, and CHMP4B) in 351 patients with endometrial cancer, and their associations with clinical, pathological, and survival outcomes were analyzed.

**Results:**

High NLRP3 expression was significantly associated with age ≤ 50 years and premenopause. Increased cleaved caspase-1 p20 expression was associated with nonendometrioid carcinoma, Federation of Gynaecology and Obstetrics (FIGO) grade 3, and the p53 mutant pattern and was independently associated with poor recurrence-free survival (RFS) and overall survival. Increased cleaved gasdermin D expression was associated with a body mass index of >25 kg/m², FIGO grades 1–2, early FIGO stage (I–II), and absence of lymph node metastasis. High CHMP4B expression was associated with nonendometrioid carcinoma and poor RFS. Cleaved gasdermin D-high/CHMP4B-low endometrial cancer was associated with endometrioid carcinoma, FIGO grades 1–2 and favorable RFS.

**Discussion:**

Our study identified cleaved caspase-1 p20 as an independent predictor of adverse outcomes in endometrial cancer. CHMP4B, an inhibitor of pyroptosis, was associated with an unfavorable RFS, whereas high cleaved gasdermin D/low CHMP4B expression was associated with a favorable RFS. These findings underscore the prognostic significance of pyroptosis and the potential interaction between cleaved gasdermin D and CHMP4B in endometrial cancer.

## 1 Introduction

Endometrial cancer remains the most prevalent malignancy of the female genital tract in developed countries, with its incidence steadily increasing (1–5). Although early-stage endometrial cancer demonstrates a favorable overall survival (OS) exceeding 80%, its prognosis drastically worsens in cases with advanced disease (stage IV), where the 5-year OS plummets to less than 20% (5, 6). Despite the recent emergence of immune checkpoint inhibitors for mismatch repair (MMR)-deficient or microsatellite instability-high endometrial cancer, targeted therapy options for the treatment of advanced or recurrent cases remain limited, with conventional chemotherapy being the primary treatment option (7).

Recently, pyroptosis, a type of nonapoptotic cell death mediated by caspase-1, has garnered attention in cancer research. *In vitro* and *in vivo* studies have shown that some pyroptosis-inducing agents demonstrate antitumor effects in various cancers, including ovarian cancer (8), hepatocellular carcinoma (9), anaplastic thyroid cancer (10), and nonsmall cell lung cancer (11).

NLR family pyrin domain-containing 3 (NLRP3), which functions as a cytosolic sensor of inflammasomes, plays a central role in responding to exogenous or endogenous danger signals by initiating the canonical pyroptotic pathway (12–14). This process involves the conversion of pro-caspase into active caspase-1, which triggers the generation of catalytically active p20/p10 cleavage fragments (12–17). Following activation, caspase-1 cleaves gasdermin D after Asp275, creating N- and C-terminal domains (12–14, 18). The N-terminal domain forms pores on the cell membrane, promoting the release of intracellular contents, such as interleukin-1β and -18, and resulting in pyroptotic cell death (13, 14, 18).

The endosomal sorting complex required for transport (ESCRT)-III machinery plays a pivotal role in various membrane remodeling and scission processes (19, 20). Charged multivesicular body protein 4B (CHMP4B) serves as a crucial component of the ESCRT-III machinery and has been demonstrated to be involved in the repair of plasma membrane damage caused by gasdermin D pore-induced calcium influx (19, 21, 22). As such, it functions as a de facto inhibitor of pyroptosis, limiting proinflammatory cytokine secretion and preventing pyroptosis-induced cell death (19, 21).

The current study aimed to assess the clinicopathologic significance of four key molecules associated with pyroptosis, which include three pivotal components within the canonical pathway (NLRP3, cleaved caspase-1 p20, and cleaved gasdermin D) and one inhibitory molecule (CHMP4B).

## 2 Materials and methods

### 2.1 Tumor samples

This study included 351 patients with endometrial cancer who underwent surgical resection between 2010 and 2022 and had available formalin-fixed, paraffin-embedded blocks. Data retrieved from electronic medical records encompassed patient demographics, including age, body mass index (BMI), menopausal status, parity, and survival outcomes. This study was approved by the institutional review board of the Gachon University Gil Medical Center (Incheon, Republic of Korea), which waived patient consent (Approval Number GBIRB 2022-236).

### 2.2 Pathologic evaluation

Information regarding the primary diagnosis and tumor size was obtained from surgical pathology reports. Hematoxylin and eosin (H&E)-stained slides underwent a thorough review to reassess various pathologic features, such as diagnosis, International Federation of Gynaecology and Obstetrics (FIGO) grade, FIGO stage, lymphovascular invasion, lymph node metastasis, MMR status, and p53 status.

### 2.3 Immunohistochemistry

Immunohistochemical staining was conducted using tissue microarray (TMA) blocks, with a single 2-mm core representing an individual patient. The blocks were subsequently sectioned into 4-μm-thick slices for staining. Tissue sections were deparaffinized at 60°C and rehydrated through sequential immersion in xylene and graded ethanol series. Endogenous peroxidase activity was blocked by incubation in 3% hydrogen peroxide for 10 min, followed by heat-induced antigen retrieval. Tissue sections were incubated overnight at 4°C with primary antibodies, including anti-NLRP3 (1:150, AG-20B-0014-C100, Adipogen, San Diego, CA, United States), anti-cleaved caspase-1 p20 (Asp296) (1:100, PA5-99390, Thermo Fisher, Waltham, MA, United States), anti-cleaved gasdermin D (Asp275) (1:500, 36425, Cell Signaling, Danvers, MA, United States), and anti-CHMP4B (1:20, polyclonal, Invitrogen, Waltham, MA, United States). Following overnight incubation, counterstaining with hematoxylin was conducted for 3 min. Subsequently, the sections were dehydrated through immersion in ethanol and xylene, followed by mounting.

**Figure 1:**
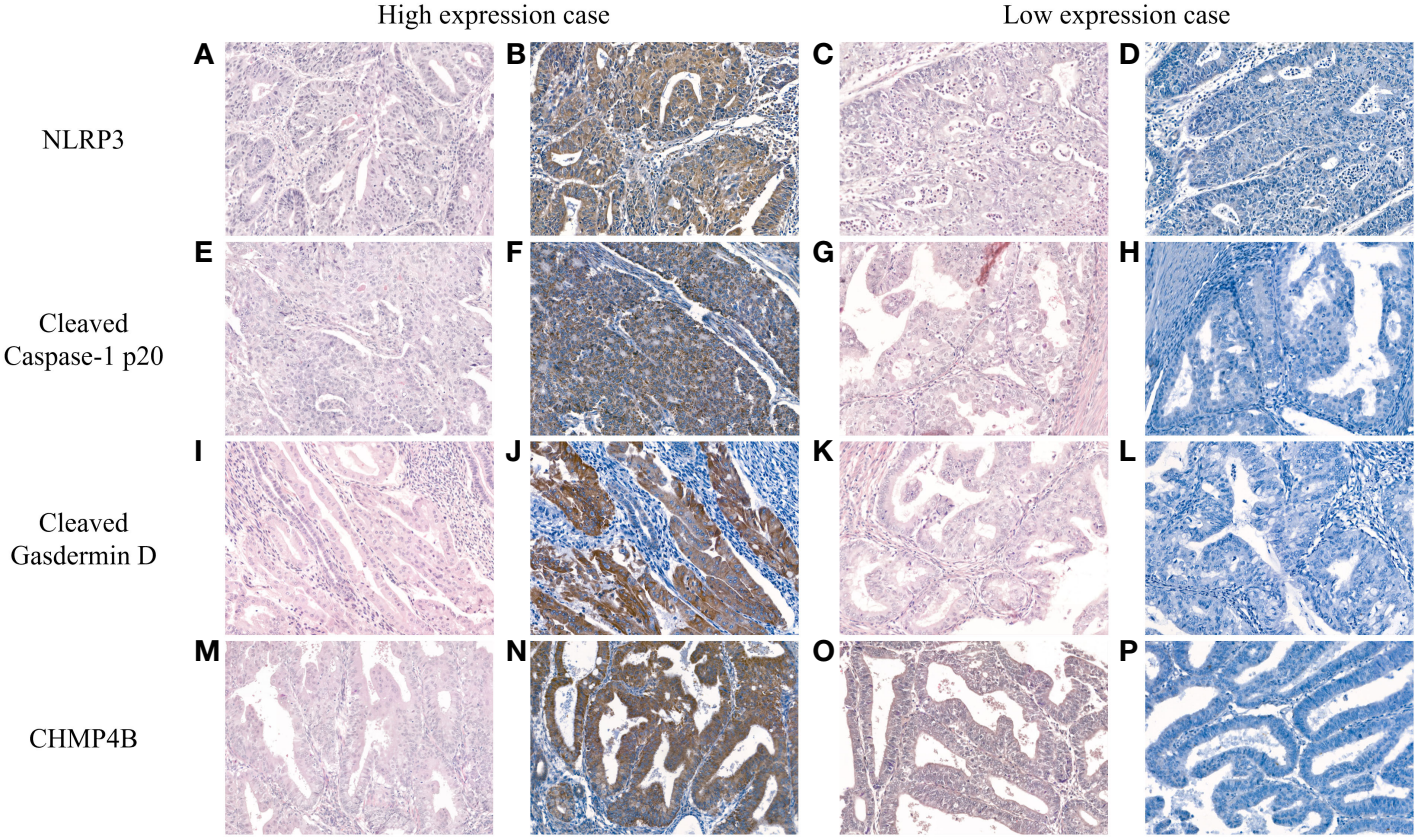
Representative hematoxylin and eosin (H&E) and immunohistochemistry images depicting high and low expression of NLRP3 **(A–D)**, cleaved caspase-1 p20 **(E–H)**, cleaved gasdermin D **(I–L)**, and CHMP4B **(M–P)** in endometrial cancer [H&E **(A**, **C**, **E**, **G**, **I**, **K**, **M**, **O)**; NLRP3 **(B**, **D)**; cleaved caspase-1 p20, **(F**, **H)**; cleaved gasdermin D **(J**, **L)**; CHMP4B **(N**, **P)**; original magnification, all ×200].

The intensities of NLRP3, cleaved caspase-1 p20, cleaved gasdermin D, and CHMP4B immunostaining slides were assessed based on the following scale: negative (0), weak positive (1+), moderate positive (2+), and strong positive (3+). Subsequently, binary classification into low and high expressions was performed. For NLRP3 and cleaved gasdermin D, negative was categorized as low expression, whereas weak to strong positive were classified as high expression. For cleaved caspase-1 p20 and CHMP4B, negative to moderate positive were designated as low expression, whereas strong positive was considered high expression. Representative images of high and low expression for each marker are illustrated in **Figure 1**.

### 2.4 Outcome measures

The primary outcomes were recurrence-free survival (RFS) and OS. RFS was defined as the duration from surgical resection to the first recurrence or last follow-up, with patients with synchronous distant metastasis being excluded from RFS analysis. OS was defined as the duration from surgical resection to death from any cause or last follow-up.

### 2.5 Statistical analysis

Continuous variables were compared using either the independent two-sample t-test or Mann–Whitney U test, whereas categorical variables were assessed using the chi-square test or Fisher exact test. Survival curves were generated using the Kaplan–Meier method and compared using the log-rank test. A univariate Cox regression analysis was conducted to identify clinicopathologic characteristics associated with RFS and OS. Additionally, multivariate Cox regression analysis was performed to identify independent prognostic significance. All statistical tests were performed using SPSS version 21 (IBM Corp., Armonk, NY, United States), with a p < 0.05 indicating statistical significance.

## 3 Results

### 3.1 Baseline characteristics of patients with endometrial cancer

**Table 1** summarizes the baseline clinical and pathologic characteristics of the included patients. Notably, the included patients had a mean age of 54.1 ± 9.8 years (range, 23–82 years), with 115 (32.8%) and 236 (67.2%) aged ≤50 and >50 years, and a mean BMI of 26.3 ± 4.9 kg/m², with 154 (43.9%) and 197 (56.1%) having a BMI of ≤25 kg/m² and >25 kg/m², respectively. Moreover, 205 (60.8%) and 132 (39.2%) patients had a postmenopausal and premenopausal status, respectively, whereas 52 (14.8%) and 299 (85.2%) patients had a nulliparous and parous status, respectively. The diagnosis included endometrioid carcinoma in 323 patients (92.0%) and nonendometrioid carcinoma in 28 patients (8.0%), which comprised 9 serous carcinomas, 4 clear cell carcinomas, 12 mixed carcinomas, 2 undifferentiated carcinomas, and 1 carcinosarcoma. Among the included patients, 135 (38.5%), 160 (45.6%), and 56 (16.0%) had FIGO grades 1, 2, and 3, respectively, whereas 283 (80.6%), 19 (5.4%), 39 (11.1%), and 10 (2.8%) were in FIGO stages 1, 2, 3, and 4, respectively. The mean tumor diameter was 3.7 ± 2.4 cm (0.1–13.0 cm). Lymphovascular invasion was present and absent in 120 (34.2%) and 231 (65.8%) patients, respectively, whereas lymph node metastasis was observed and absent in 35 (90.0%) and 316 (10.0%) patients, respectively. Defective MMR, indicated by the absence of nuclear staining in the four MMR proteins (MLH1, MSH2, MSH6, and PMS2), was detected in 74 cases (21.1%), whereas normal MMR was observed in 274 cases (78.1%). P53 status, determined through p53 immunohistochemistry, exhibited a mutant pattern (including a null-mutant pattern) in 52 (14.8%) and a wild-type pattern in 292 (83.2%) patients. The median follow-up duration was 53 months (range, 1–78 months).

**Table 1:** Baseline clinicopathologic characteristics of patients with endometrial cancer.

Patient characteristics	N (%)
Age	
≤50	115 (32.8)
>50	236 (67.2)
Body mass index (kg/m²)	
≤25	154 (43.9)
>25	197 (56.1)
Menopausal status	
Premenopausal	132 (39.2)
Postmenopausal	205 (60.8)
Parity	
Nulliparous	52 (14.8)
Parous	299 (85.2)
Diagnosis	
Endometrioid carcinoma	323 (92.0)
Nonendometrioid carcinoma	28 (8.0)
FIGO grade	
Grades 1–2	295 (84.0)
Grade 3	56 (16.0)
FIGO stage	
Early stage (I–II)	302 (86.0)
Advanced stage (III–IV)	49 (14.0)
Lymphovascular invasion	
Absent	231 (65.8)
Present	120 (34.2)
Lymph node metastasis	
Absent	316 (90.0)
Present	35 (10.0)
Mismatch repair status	
Normal	274 (78.7)
Defective	74 (21.3)
P53 status	
Wild-type pattern	292 (84.9)
Mutant pattern	52 (15.1)

### 3.2 Expression of four pyroptosis-related markers and clinicopathologic correlation

Immunohistochemistry revealed high and low NLRP3 expression in 235 (67.0%) and 115 (32.8%) patients, high and low cleaved caspase-1 p20 expression in 11 (3.1%) and 333 (94.9%) patients, high and low cleaved gasdermin D expression in 49 (14.0%) and 300 (85.5%) patients, and high and low CHMP4B expression in 9 (2.6%) and 340 (96.9%) patients, respectively.

The correlation between clinicopathologic characteristics and the expression of the four pyroptosis-related markers is presented in **Table 2**. Notably, our findings showed that high NLRP3 expression was associated with age ≤ 50 years (p = 0.022) and premenopausal status (p = 0.012); high cleaved caspase-1 p20 expression was associated with nonendometrioid carcinoma (p = 0.008), FIGO grade 3 disease (p = 0.020), and a p53 mutant pattern (p = 0.003); high cleaved gasdermin D expression was associated with BMI > 25 kg/m² (p = 0.049), FIGO grades 1–2 (p = 0.014), early FIGO stage (I–II) (p = 0.030), and absence of lymph node metastasis (p = 0.042); and high CHMP4B expression was associated with nonendometrioid carcinoma (p = 0.028).

**Table 2:** Correlation between pyroptosis-related markers and clinicopathologic characteristics.

Patient characteristics	NLRP3			Cleaved caspase-1 p20			Cleaved gasdermin D			CHMP4B		
	Low	High	p value	Low	High	p value	Low	High	p value	Low	High	p value
Age			0.022			0.513			0.962			0.481
≤50	28 (24.3)	86 (36.6)		108 (32.4)	2 (18.2)		99 (33.0)	16 (32.7)		110 (32.4)	4 (44.4)	
>50	87 (75.7)	149 (63.4)		225 (67.6)	9 (81.8)		201 (67.0)	33 (67.3)		230 (67.6)	5 (55.6)	
Body mass index (kg/m²)			0.313			0.221			0.049			0.737
≤25	55 (47.8)	99 (42.1)		143 (42.9)	7 (63.6)		137 (45.7)	15 (30.6)		150 (44.1)	3 (33.3)	
>25	60 (52.2)	136 (57.9)		190 (57.1)	4 (36.4)		163 (54.3)	34 (69.4)		190 (55.9)	6 (66.7)	
Menopausal status			0.012			0.214			0.078			0.999
Premenopausal	32 (29.4)	99 (43.6)		125 (39.2)	2 (18.2)		108 (37.5)	24 (51.1)		128 (39.1)	3 (37.5)	
Postmenopausal	77 (70.6)	128 (56.4)		194 (60.8)	9 (81.8)		180 (62.5)	23 (48.9)		199 (60.9)	5 (62.5)	
Parity			0.323			0.380			0.762			0.999
Nulliparous	14 (12.2)	38 (16.2)		51 (15.3)	0 (0)		44 (14.7)	8 (16.3)		51 (15.0)	1 (11.1)	
Parous	101 (87.8)	197 (83.7)		282 (84.7)	11 (100)		256 (85.3)	41 (83.7)		289 (85.0)	8 (88.9)	
Diagnosis			0.356			0.008			0.151			0.028
Endometrioid carcinoma	108 (93.9)	214 (91.1)		309 (92.8)	7 (63.6)		273 (91.0)	48 (98.0)		315 (92.6)	6 (66.7)	
Nonendometrioid carcinoma	7 (6.1)	21 (8.9)		24 (7.2)	4 (36.4)		27 (9.0)	1 (2.0)		25 (7.4)	3 (33.3)	
FIGO grade			0.901			0.020			0.014			0.162
Grades 1–2	97 (84.3)	197 (83.8)		282 (84.7)	6 (54.5)		246 (82.0)	47 (95.9)		287 (84.4)	6 (66.7)	
Grade 3	18 (15.7)	38 (16.2)		51 (15.3)	5 (45.5)		54 (18.0)	2 (4.1)		53 (15.6)	3 (33.3)	
FIGO stage			0.718			0.056			0.030			0.368
Early stage (I–II)	100 (87.0)	201 (85.5)		288 (86.5)	7 (63.6)		253 (84.3)	47 (95.9)		293 (86.2)	7 (77.8)	
Advanced stage (III–IV)	15 (13.0)	34 (14.5)		45 (13.5)	4 (36.4)		47 (15.7)	2 (4.1)		47 (13.8)	2 (22.2)	
Lymphovascular invasion			0.273			0.999			0.783			0.724
Absent	71 (61.7)	159 (67.7)		218 (65.5)	7 (63.6)		196 (65.3)	33 (67.3)		222 (65.3)	7 (77.8)	
Present	44 (38.3)	76 (32.3)		115 (34.5)	4 (36.4)		104 (3407)	16 (32.7)		118 (34.7)	2 (22.2)	
Lymph node metastasis			0.184			0.310			0.042			0.999
Absent	100 (87.0)	215 (91.5)		300 (90.1)	9 (81.8)		266 (88.7)	48 (98.0)		306 (90.0)	8 (88.9)	
Present	15 (13.0)	20 (8.5)		33 (9.9)	2 (18.2)		34 (11.3)	1 (2.0)		34 (10.0)	1 (11.1)	
Mismatch repair status			0.895			0.468			0.578			0.690
Normal	90 (78.3)	183 (78.9)		257 (77.9)	10 (90.9)		232 (78.1)	40 (81.6)		264 (78.3)	8 (88.9)	
Defective	25 (21.7)	49 (21.1)		73 (22.1)	1 (9.1)		65 (21.9)	9 (18.4)		73 (21.7)	1 (11.1)	
P53 status			0.466			0.003			0.056			0.631
Wild-type pattern	99 (86.8)	192 (83.8)		280 (85.9)	5 (45.5)		244 (83.3)	46 (93.9)		283 (85.0)	7 (77.8)	
Mutant pattern	15 (13.2)	37 (16.2)		46 (14.1)	6 (54.5)		49 (16.7)	3 (6.1)		50 (15.0)	2 (22.2)	

The correlation between the expression of the four pyroptosis-related markers is presented in **Table 3**. Accordingly, our results showed that high NLRP3 expression was associated with high cleaved caspase-1 p20 expression (p = 0.019) and that high cleaved caspase-1 p20 expression was associated with high CHMP4B expression (p = 0.030).

**Table 3:** Correlation between pyroptosis-related markers.

	NLRP3			Cleaved caspase-1 p20			Cleaved gasdermin D			CHMP4B		
	Low	High	p value	Low	High	p value	Low	High	p value	Low	High	p value
NLRP3			NA			0.019			0.673			0.281
Low	NA	NA		112 (33.7)	0 (0)		97 (32.3)	17 (35.4)		113 (33.3)	1 (11.1)	
High	NA	NA		220 (66.3)	11 (100)		203 (67.7)	31 (64.6)		226 (66.7)	8 (88.9)	
Cleaved caspase-1 p20			0.019			NA			0.652			0.030
Low	112 (100)	220 (95.2)		NA	NA		287 (97.0)	45 (95.7)		326 (97.3)	7 (77.8)	
High	0 (0)	11 (4.8)		NA	NA		9 (3.0)	2 (4.3)		9 (2.7)	2 (22.2)	
Cleaved gasdermin D			0.673			0.652			NA			0.620
Low	97 (85.1)	203 (86.8)		287 (86.4)	9 (81.8)		NA	NA		290 (85.5)	9 (100)	
High	17 (14.9)	31 (13.2)		45 (13.6)	2 (18.2)		NA	NA		49 (14.5)	0 (0)	
CHMP4B			0.281			0.030			0.620			NA
Low	113 (99.1)	226 (96.6)		326 (97.9)	9 (81.8)		290 (97.0)	49 (100)		NA	NA	
High	1 (0.9)	8 (3.4)		7 (2.1)	2 (18.2)		9 (3.0)	0 (0)		NA	NA	

To evaluate the consequences of cleaved gasdermin D-mediated pyroptosis without CHMP4B-mediated membrane repair, we analyzed the clinicopathologic characteristics of cleaved gasdermin D-low/CHMP4B-high and cleaved gasdermin D-high/CHMP4B-low endometrial cancer (**Supplementary Table 1**). Notably, cleaved gasdermin D-high/CHMP4B-low endometrial cancer was associated with endometrioid carcinoma (p = 0.010) and FIGO grade 1–2 (p = 0.023), whereas cleaved gasdermin D-high/CHMP4B-low endometrial cancer was significantly associated with endometrioid carcinoma (p = 0.010) and FIGO grades 1–2 (p = 0.023).

### 3.3 Univariate survival analysis for pyroptosis-related markers

**Figure 2:**
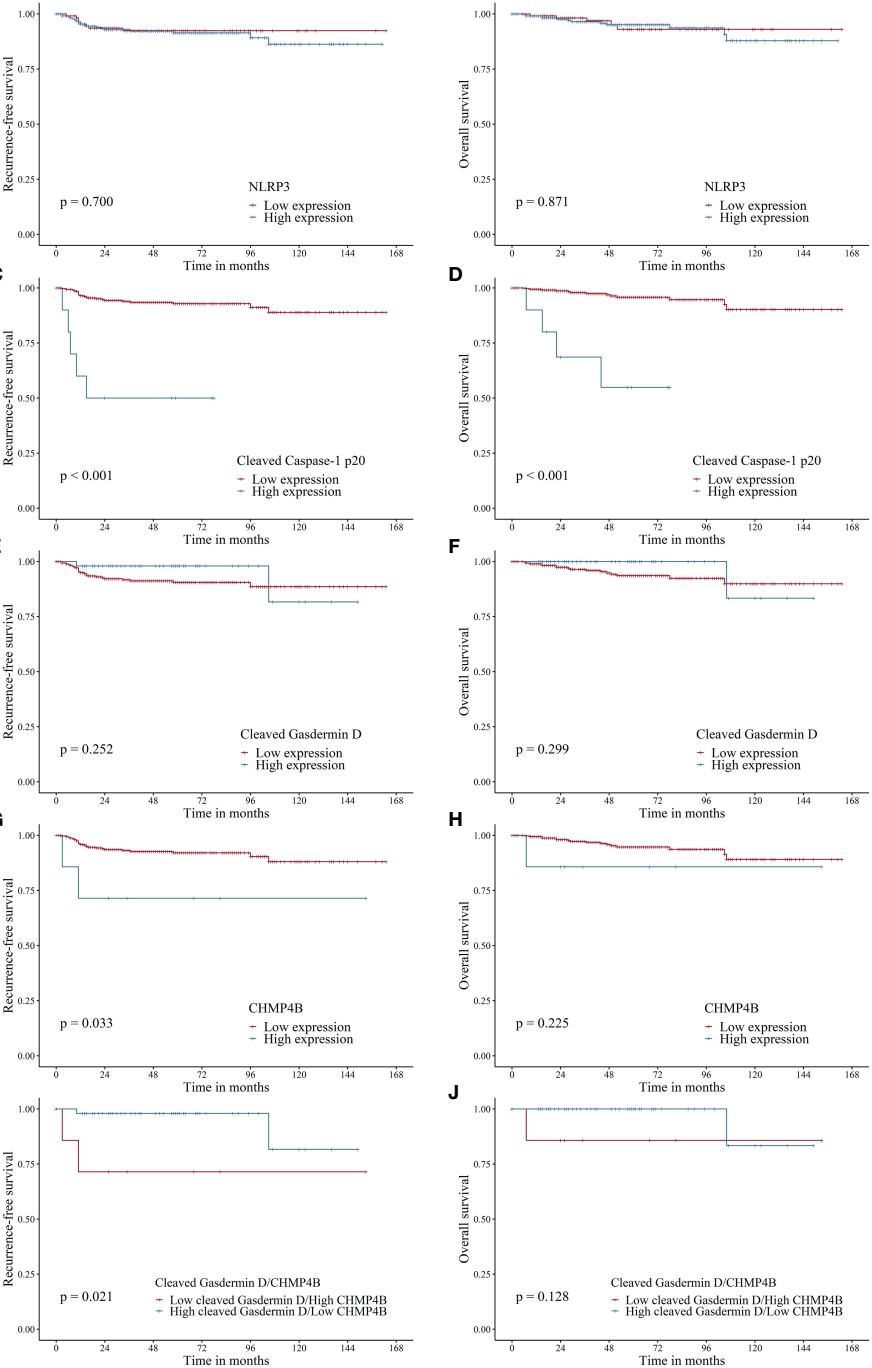
Recurrence-free survival (RFS) and overall survival (OS) in patients with endometrial cancer based on NLRP3, cleaved caspase-1 p20, cleaved gasdermin D, and CHMP4B expression. **(A, B)**. NLRP3 expression was not associated with RFS (p = 0.700) and OS (p = 0.871). **(C, D)**. High cleaved caspase-1 p20 was associated with adverse RFS (p < 0.001) and OS (p < 0.001). **(E, F)**. Cleaved gasdermin D was not associated with RFS (p = 0.252) and OS (p = 0.299). **(G, H)**. High CHMP4B expression was associated with adverse RFS (p = 0.033) but not OS (p = 0.225). **(I, J)**. High cleaved gasdermin D/low CHMP4B was associated with favorable RFS (p = 0.021) but not OS (p = 0.128) when compared to low cleaved gasdermin D/high CHMP4B.

After evaluating the correlation between pyroptosis-related marker expression and survival (RFS and OS), we found that high NLRP3 expression was not associated with RFS (HR, 1.176; 95% CI, 0.514–2.691; log-rank p = 0.070) and OS (HR, 1.091; 95% CI, 0.383–3.108; log-rank p = 0.871) (**Table 4**, **Figures 2A, B**). However, high cleaved caspase-1 p20 expression was associated with worse RFS (HR, 11.359; 95% CI, 4.253–30.334; log-rank p < 0.001) and OS (HR, 1.091; 95% CI, 0.383–3.108; log-rank p < 0.001) (**Table 4**, **Figures 2C, D**). Moreover, high cleaved gasdermin D expression was not associated with RFS (HR, 0.442; 95% CI, 0.105–1.865; log-rank p = 0.252) and OS (HR, 0.359; 95% CI, 0.048–2.708; log-rank p = 0.299) (**Table 4**, **Figures 2E, F**). High CHMP4B expression was associated with worse RFS (HR, 4.220; 95% CI, 0.999–17.827; log-rank p = 0.033) but not with OS (HR, 3.260; 95% CI, 0.432–24.623; log-rank p = 0.225) (**Table 4**, **Figures 2G, H**).

**Table 4:** Univariate survival analysis on the association between clinicopathologic factors and recurrence or death in patients with endometrial cancer.

Patient characteristics	Recurrence-free survival			Overall survival		
	HR	95% CI	p value	HR	95% CI	p value
Age > 50	3.925	1.182–13.038	0.026	1.529	0.498–4.693	0.458
Body mass index (kg/m²) > 25	1.136	0.526–2.457	0.745	0.683	0.260–1.792	0.438
Postmenopause	3.487	1.197–10.160	0.022	1.93	0.622–5.985	0.255
Parous	2.363	0.560–9.979	0.242	2.946	0.390–22.255	0.295
Nonendometrioid carcinoma	7.964	3.568–17.776	<0.001	7.25	2.669–19.693	<0.001
Grade 3	5.472	2.571–11.646	<0.001	3.678	1.399–9.669	0.008
Advanced stage (III–IV)	5.069	2.318–11.084	<0.001	8.356	3.212–21.740	<0.001
Lymphovascular invasion	3.280	1.522–7.071	0.002	2.411	0.930–6.253	0.070
Lymph node metastasis	2.837	1.143–7.042	0.025	4.718	1.728–12.882	0.002
Defective MMR	0.856	0.324–2.261	0.754	0.537	0.123–2.352	0.410
Mutant pattern p53	4.628	2.136–10.024	<0.001	3.814	1.401–10.379	0.009
High NLRP3	1.176	0.514–2.691	0.701	1.091	0.383–3.108	0.871
High cleaved caspase-1 p20	11.359	4.253–30.334	<0.001	14.791	4.685–46.698	<0.001
High cleaved gasdermin D	0.442	0.105–1.865	0.266	0.359	0.048–2.708	0.320
High CHMP4B	4.220	0.999–17.827	0.050	3.260	0.432–24.623	0.252

To evaluate the impact of cleaved gasdermin D-mediated pyroptosis unhindered by CHMP4B, we compared survival outcomes between cleaved gasdermin D-low/CHMP4B-high and cleaved gasdermin D-high/CHMP4B-low endometrial cancers. Accordingly, we found that cleaved gasdermin D-high/CHMP4B-low cancer was significantly associated with favorable RFS (log-rank p = 0.021) and tended to have a favorable OS (log-rank p = 0.128) (**Figures 2I, J**).

We then performed subgroup analysis among patients with endometrial cancer who had advanced disease stages (FIGO III–IV). Notably, among those with advanced stage, high NLRP3 was associated with adverse RFS (log-rank p = 0.009) but not OS (log-rank p = 0.357) (**Supplementary Figures 1A, B**), high cleaved caspase-1 p20 expression was associated with worse RFS (log-rank p < 0.001) and OS (log-rank p < 0.001) (**Supplementary Figures 1C, D**), high cleaved gasdermin D expression was not associated with RFS (log-rank p = 0.464) and OS (log-rank p = 0.580) (**Supplementary Figures 1E, F**), and high CHMP4B was not associated with RFS (log-rank p = 0.164) and OS (log-rank p = 0.057) (**Supplementary Figures 1G, H**).

### 3.4 Univariate survival analysis for other clinicopathologic factors

Other clinicopathologic factors found to be associated with RFS included age > 50 years (HR, 3.925; 95% CI, 1.182–13.038), postmenopausal status (HR, 3.487; 95% CI, 1.197–10.160), nonendometrioid carcinoma (HR, 7.964; 95% CI, 3.568–17.776), FIGO grade 3 (HR, 5.472; 95% CI, 2.571–11.646), advanced stage (FIGO stages III–IV) (HR, 5.069; 95% CI, 2.318–11.084), lymphovascular invasion (HR, 3.280; 95% CI, 1.522–7.071), lymph node metastasis (HR, 2.837; 95% CI, 1.143–7.042), and p53 mutant pattern (HR, 4.628; 95% CI, 2.136–10.024) (**Table 4**). Other clinicopathologic factors found to be associated with OS included nonendometrioid carcinoma (HR, 7.250; 95% CI, 2.669–19.693), FIGO grade 3 (HR, 3.678; 95% CI, 1.399–9.669), advanced stage (FIGO stages III–IV) (HR, 8.356; 95% CI, 3.212–21.740), lymph node metastasis (HR, 4.718; 95% CI, 1.728–12.882), and p53 mutant pattern (HR, 3.814; 95% CI, 1.401–10.379) (**Table 4**).

### 3.5 Multivariate survival analysis for a pyroptosis-related marker

Given that high cleaved caspase-1 p20 expression was significantly associated with RFS and OS on univariate Cox regression, it was incorporated into the multivariate Cox regression model. Other clinicopathologic characteristics significantly associated with RFS and OS on univariate analysis were adjusted during multivariate analysis. Notably, high cleaved caspase-1 p20 expression (HR, 7.017; 95% CI, 2.054–23.975) and lymphovascular invasion (HR, 3.067; 95% CI, 1.161–8.103) were identified as independent prognostic factors for adverse RFS (**Table 5**), whereas high cleaved caspase-1 p20 expression (HR, 5.289; 95% CI, 1.270–22.033) and advanced stage (FIGO stages III–IV) (HR, 6.525;95% CI, 1.586–26.838) were identified as independent prognostic factors for adverse OS (**Table 5**).

**Table 5:** Multivariate survival analysis on the association between clinicopathologic factors and recurrence or death in patients with endometrial cancer.

Patient characteristics	Recurrence-free survival			Overall survival		
	HR	95% CI	p value	HR	95% CI	p value
Age > 50	1.598	0.170–15.042	0.682	NA	NA	NA
Postmenopause	1.279	0.172–9.486	0.810	NA	NA	NA
Nonendometrioid carcinoma	2.723	0.805–9.211	0.107	3.253	0.511–20.712	0.212
Grade 3	1.322	0.419–4.174	0.634	0.613	0.101–3.724	0.595
Advanced stage (III–IV)	2.127	0.625–7.233	0.227	6.525	1.586–26.838	0.009
Lymphovascular invasion	3.067	1.161–8.103	0.024	NA	NA	NA
Lymph node metastasis	0.570	0.153–2.122	0.402	0.779	0.200–3.039	0.719
Mutant pattern p53	1.583	0.602–4.165	0.352	1.278	0.362–4.516	0.704
High cleaved Caspase-1 p20	7.017	2.054–23.975	0.002	5.289	1.270–22.033	0.022

## 4 Discussion

The current study successfully identified a novel prognostic biomarker within the key molecules of the pyroptosis pathway. Accordingly, our findings showed that cleaved caspase-1 p20, an effector protein within the inflammasome of the canonical pyroptosis pathway (14), was independently associated with adverse RFS and OS when highly expressed in endometrial cancer. Moreover, high cleaved caspase-1 p20 expression significantly correlated with nonendometrioid carcinoma, high FIGO grade, and the presence of a p53 mutant pattern. Although the prognostic impact of caspase-1 (CASP1) has not been previously evaluated in endometrial cancer, diverse associations have been reported between this molecule and prognosis in other cancer types. For instance, high CASP1 mRNA expression has been associated with favorable outcomes in colorectal and stomach cancers, yet it correlates with adverse OS in acute myeloid leukemia (23–25). Given that caspase-1 is initially expressed as inactive pro-caspase-1 and requires subsequent activation to engage in downstream pathways leading to gasdermin D-mediated pyroptosis (13, 14, 26, 27), prior studies relying solely on mRNA expression may have failed to accurately reflect the active caspase-1 levels. Therefore, the significance of our study lies in evaluating the protein expression of cleaved caspase-1 p20 and its association with prognosis, providing a more comprehensive understanding of the intricate mechanisms involved in pyroptosis. This approach aims to overcome the constraints of mRNA-based analyses in order to provide valuable insights into the biology of pyroptosis and its implications for endometrial cancer prognosis.

Our findings revealed that high cleaved caspase-1 p20 expression was associated with high NLRP3 expression, indicative of the canonical pyroptotic pathway where caspase-1 is activated, leading to the production of cleaved caspase-1 p20 by NLRP3 (13, 14). Furthermore, our investigation revealed that high expression of NLRP3, the initiator of the canonical pyroptotic pathway responsible for activating caspase-1, was linked to adverse RFS, specifically in advanced endometrial cancer, although not across all stages of endometrial cancer. In support of these findings, Liu et al. (28) demonstrated that NLRP3 overexpression enhanced *in vitro* proliferation, migration, and invasion of endometrial cancer cells and increased active caspase-1 (cleaved caspase-1 p10), whereas *in vivo* knockdown of NLRP3 inhibited the growth of implanted endometrial cancer. These findings provide mechanistic insights into the adverse prognostic impact of high NLRP3 expression. Additionally, our results indicated that high NLRP3 expression was associated with age ≤ 50 years and premenopausal status. This association aligns with the findings of a previous study that showed upregulation of NLRP3 by estradiol (E2) in an estrogen receptor β-dependent manner (28) given that serum estradiol levels decrease dramatically in postmenopausal women (29).

The current study also found that cleaved gasdermin D, a product of active caspase-1 (13, 14), was associated with lower FIGO grade (1–2), early FIGO stage (I–II), and the absence of lymph node metastasis. Although none of our patients with high cleaved gasdermin D expression experienced recurrence or death within the 96-month follow-up period, statistical significance was not achieved. Conversely, high CHMP4B expression was linked to nonendometrioid carcinoma and adverse RFS. CHMP4B, which reverses gasdermin D-mediated pyroptosis in endometrial cancer by remodeling cell membranes, was also associated with adverse OS in the TCGA-UCEC dataset when mRNA expression was high (30).

To investigate the potential interaction between cleaved gasdermin D and CHMP4B, we conducted a comparative analysis between cleaved gasdermin D-high/CHMP4B-low endometrial cancer and cleaved gasdermin D-low/CHMP4B-high endometrial cancer. Notably, our results revealed that cleaved gasdermin D-high/CHMP4B-low endometrial cancer was significantly associated with endometrioid carcinoma, FIGO grades 1–2, and favorable RFS. These findings not only underscore the favorable prognostic impact of cleaved gasdermin D-mediated pyroptotic cancer cell death but also support the interplay between cleaved gasdermin D and the membrane repair mechanism facilitated by CHMP4B.

In light of our findings, where gasdermin D-high/CHMP4B-low, indicative of active pyroptosis (19–22), was associated with favorable RFS, one might anticipate a similar favorable prognostic impact for cleaved caspase-1 p20. This key pyroptosis molecule plays a crucial role in cleaving gasdermin D to form membrane pores, ultimately leading to cell death (13, 14, 18). However, contrary to expectations, high cleaved caspase-1 p20 expression was independently associated with adverse RFS. To provide a plausible explanation for the unexpected observation, we conducted an in-depth review of the literature, focusing on the structural attributes of caspase-1 p20.

Full-length pro-caspase-1 (p46) comprises distinct segments, including the caspase-recruitment domain (CARD), CARD domain linker (CDL), p20 segment, interdomain linker (IDL), and p10 domain, arranged from the N-terminal to the C-terminal (31). Autoproteolytic cleavage within linker residues generates two caspase-1 segments, namely p20 and p10 (17, 32). Dimeric forms of caspase-1 p20 and p10 segments, such as heterotetramer (p20_2_p10_2_) or homodimer of p20p10 heterodimers ((p20p10)_2_), are considered active conformers due to their catalytic activity, with the p20 segment containing the catalytic cysteine 285 residue (15–17, 33, 34). According to the traditional theory, high cleaved caspase-1 p20 expression would promote gasdermin D-induced pyroptosis (15, 17, 33, 34). However, recent challenges to this theory have prompted the proposal of an alternative view, suggesting that p20p10 formation leads to an unstable status, terminating protease activity (35). Instead, a transient p33p10 heterodimer and full-length p46 have been considered major and minor active conformers, respectively (35). According to this updated theory, high cleaved caspase-1 p20 expression could indicate an inactive status of gasdermin D-induced pyroptosis. This theoretical perspective offers valuable insights into reconciling the divergent prognostic implications of high cleaved caspase-1 p20, which indicates a worse prognosis, with the more favorable outlook associated with high cleaved gasdermin D and low CHMP4B expressions in endometrial cancer.

In conclusion, the current study unveiled the prognostic impact of pyroptosis-related markers in endometrial cancer. Notably, high expression of cleaved caspase-1 p20 was identified as an independent prognostic factor for adverse RFS and OS. Additionally, our findings showed that CHMP4B, recognized as an inhibitor of pyroptosis (19–22), was associated with adverse RFS, whereas the presence of high cleaved gasdermin D/low CHMP4B expression was linked to favorable RFS. These findings suggest that pyroptosis had a favorable prognostic impact on endometrial cancer, highlighting the potential interaction between cleaved gasdermin D and CHMP4B in pyroptotic cancer cell death.

## Data availability statement

The raw data supporting the conclusions of this article will be made available by the authors, without undue reservation.

## Ethics statement

The studies involving humans were approved by Institutional Review Board of the Gachon University Gil Medical Center. The studies were conducted in accordance with the local legislation and institutional requirements. The ethics committee/institutional review board waived the requirement of written informed consent for participation from the participants or the participants’ legal guardians/next of kin because written informed consent was waived due to posing no more than minimal risk to patients due to retrospective study design using formalin-fixed paraffin-embedded tissue acquired during surgical treatment.

## Author contributions

S-CH: Writing – original draft, Software, Resources, Methodology, Formal analysis, Data curation. YP: Writing – original draft, Resources, Methodology, Investigation, Formal analysis, Data curation. JK: Writing – review & editing, Visualization, Validation, Supervision, Project administration, Investigation, Funding acquisition, Conceptualization, Writing – original draft, Software, Resources, Methodology, Formal analysis, Data curation.
