# Supplementary material for: Prognostic significance of pyroptosis-associated molecules in endometrial cancer: a comprehensive immunohistochemical analysis
Source: Front Oncol. 2024 Mar 18;14:1359881. doi: 10.3389/fonc.2024.1359881 (PMC10982380; doi:10.3389/fonc.2024.1359881)
Supplement: Supplementary file 1 [file DataSheet_1.docx]

Supplementary Material

**Supplementary Table 1.** Clinicopathologic characteristics according to the combined expression of cleaved gasdermin D and CHMP4B

| Patient characteristics | Cleaved GSDMD and CHMP4B combination | | |
| --- | --- | --- | --- |
|  | Cleaved gasdermin D-low/CHMP4B high | Cleaved gasdermin D-high/CHMP4B low | p value |
| Age |  |  | 0.704 |
| ≤50 | 4 (44.4) | 16 (32.7) |  |
| >50 | 5 (55.6) | 33 (67.3) |  |
| Body mass index (kg/m²) |  |  | 0.999 |
| ≤25 | 3 (33.3) | 15 (30.6) |  |
| >25 | 6 (66.7) | 34 (69.4) |  |
| Menopausal status |  |  | 0.705 |
| Premenopausal | 3 (37.5) | 24 (51.1) |  |
| Postmenopausal | 5 (62.5) | 23 (48.9) |  |
| Parity |  |  | 0.999 |
| Nulliparous | 1 (11.1) | 8 (16.3) |  |
| Parous | 8 (88.9) | 41 (83.7) |  |
| Diagnosis |  |  | 0.010 |
| Endometrioid carcinoma | 6 (66.7) | 48 (98.0) |  |
| Nonendometrioid carcinoma | 3 (33.3) | 1 (2.0) |  |
| FIGO grade |  |  | 0.023 |
| Grade 1–2 | 6 (66.7) | 47 (95.9) |  |
| Grade 3 | 3 (33.3) | 2 (4.1) |  |
| FIGO stage |  |  | 0.110 |
| Early stage (I–II) | 7 (77.8) | 47 (95.9) |  |
| Advanced stage (III–IV) | 2 (22.2) | 2 (4.1) |  |
| Lymphovascular invasion |  |  | 0.706 |
| Absent | 7 (77.8) | 33 (67.3) |  |
| Present | 2 (22.2) | 16 (32.7) |  |
| Lymph node metastasis |  |  | 0.289 |
| Absent | 8 (88.9) | 48 (98.0) |  |
| Present | 1 (11.1) | 1 (2.0) |  |
| Mismatch repair status |  |  | 0.999 |
| Normal | 8 (88.9) | 40 (81.6) |  |
| Defective | 1 (11.1) | 9 (18.4) |  |
| P53 status |  |  | 0.168 |
| Wild-type pattern | 7 (77.8) | 46 (93.9) |  |
| Mutant pattern | 2 (22.2) | 3 (6.1) |  |

**
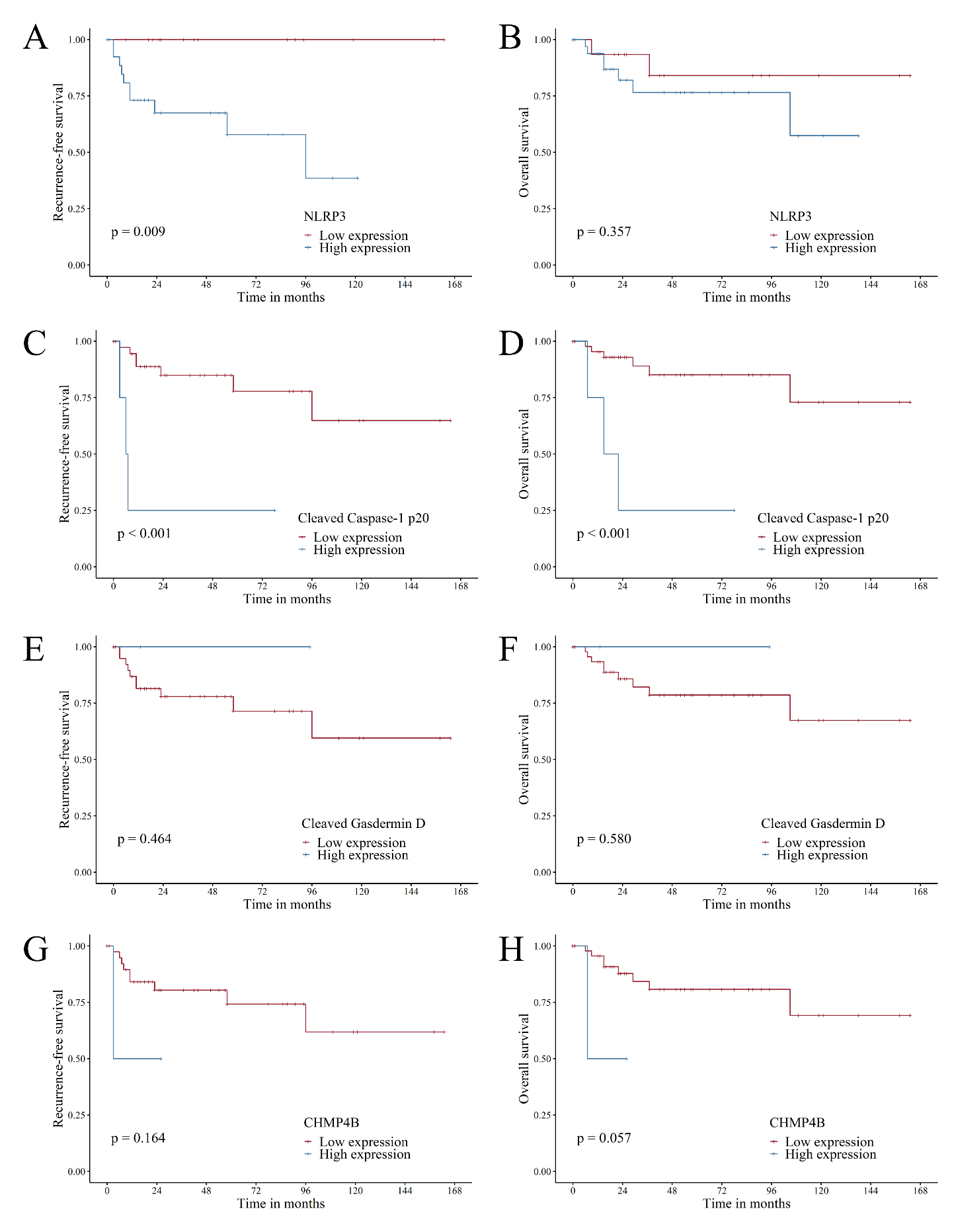
**

**Supplementary Figure 1.** Recurrence-free survival (RFS) and overall survival (OS) in patients with advanced FIGO stage (III–IV) endometrial cancer based on NLRP3, cleaved caspase-1 p20, cleaved gasdermin D, and CHMP4B expression. A–B. NLRP3 expression was associated with adverse RFS (p = 0.009) but not OS (p = 0.357). C–D. High cleaved caspase-1 p20 was linked to adverse RFS (p < 0.001) and OS (p < 0.001). E–F. Cleaved gasdermin D was not associated with RFS (p = 0.464) and OS (p = 0.580). G–H. High CHMP4B expression was not significantly associated with RFS (p = 0.164) and OS (p = 0.057).
